# Preoperative Vitamin.D Status and Post-Total Thyroidectomy Hypocalcemia

**DOI:** 10.22038/IJORL.2023.75069.3518

**Published:** 2024-01

**Authors:** Parvin Layegh, Zakie Sadat Sajjadi, Leila V. Mostaan, Masoud Mohebbi, Mona Kabiri, Mohammad Ali Yaghoubi

**Affiliations:** 1 *Metabolic Syndrome Research Center, Mashhad University of Medical Sciences, Mashhad, Iran. *; 2 *Department of Otorhinolaryngology, Imam Reza Hospital, Mashhad University of Medical Sciences, Mashhad, Iran.*; 3 *Cancer Research Center, Mashhad University of Medical Sciences, Mashhad, Iran.*; 4 *Nanotechnology Research Center, Pharmaceutical Technology Institute, Mashhad University of Medical Sciences, Mashhad, Iran.*

**Keywords:** Hypocalcemia, Vitamin.D, Total thyroidectomy

## Abstract

**Introduction:**

Hypocalcemia is a common complication of total thyroidectomy (TT). This study was designed to investigate the effect of preoperative vitamin.D (Vit.D) status on the occurrence of post-total thyroidectomy hypocalcemia.

**Materials and Methods:**

Patients who underwent TT without parathyroidectomy were divided into three groups based on their preoperative Serum Vit.D levels (<20 ng/ml, 20-30 ng/ml, and ≥30 ng/ml were considered deficient, insufficient, and normal Vit.D levels, respectively). Serum levels of calcium and phosphorus were measured before and 24 hours after surgery in all patients. The patients were examined for clinical symptoms and signs of hypocalcemia postoperatively. In cases with positive clinical symptoms and signs of hypocalcemia and/or calcium levels <8 mg/dl, PTH level was measured before starting calcium infusion, while serum calcium and phosphorus levels were also measured 24 hours later.

**Results:**

Among 100 patients enrolled in this study, 81% were females. The mean age was 36.60±8.32 years. Before surgery, the mean Vit.D level was 26.9±16.89 ng/ml, while 47% of cases had normal Vit.D level, 32% had insufficient vitamin levels, and 21% had Vit.D deficiency. Twenty-four hours after surgery, the calcium (P=0.356) and phosphorus (P=0.743) levels were not significantly different between the three Vit.D groups. A comparison of postoperative PTH levels between the three Vit.D groups showed no significant difference (P=0.596).

**Conclusions:**

Based on our findings, preoperative serum Vit.D levels did not affect postoperative serum calcium levels.

## Introduction

Total thyroidectomy (TT) is one of the most common head and neck surgeries in the world, and post-thyroidectomy hypocalcemia is an important complication that can lead to serious consequences and even death (1,2). The incidence of hypocalcemia after TT is reported to be about 1.2- 40% (3). The most common cause of hypocalcemia after TT is hypoparathyroidism. Other factors such as advanced age, gender, surgical technique, surgeon's expertise, autoimmune thyroid disease, concurrent lymph node dissection, maximum diameter of the thyroid, the need for subsequent surgery, and preoperative blood calcium levels also play significant roles and influence outcomes (4-6). On the other hand, the active form of Vitamin.D (Vit.D) and PTH are necessary for regulating calcium homeostasis (7). Serum Vit.D (25(OH) D) level before surgery is suggested as a risk factor for the occurrence of hypocalcemia after TT (8-11). Despite many studies to evaluate the effect of Vit.D deficiency on hypocalcemia after TT, the results are still contradictory and there is no consensus among researchers (12,13). The estimated incidence of Vit.D deficiency in Iran was 56% in the study by Vatandost et al. They showed that 64% of women and 40% of men suffer from Vit.D deficiency (14). So, concerning the high incidence of Vit.D deficiency in our country, the present study was planned to evaluate the role of Vit.D status before TT on hypocalcemia after surgery.

## Materials and Methods

Among 100 patients aged 18-85 years who had undergone TT for malignant and non-malignant causes were enrolled in this study. Patients with conditions that affect Vit.D, calcium, and albumin levels, such as chronic kidney failure, malabsorption, parathyroidectomy, and drug therapy for osteoporosis, were excluded. Patients were divided into three groups based on the Vit.D level before surgery: Vit.D levels <20 ng/ml (Vit.D deficiency), 20-30 ng/ml (Vit.D Insufficiency), and ≥30 ng/ml (normal Vit.D level). Calcium and phosphorus levels were measured before and 24 hours after surgery. The patients were examined for clinical symptoms and signs of hypocalcemia postoperatively. In cases with positive clinical symptoms and signs of hypocalcemia and/or calcium levels <8 mg/dl, PTH level was measured before starting calcium infusion, while serum calcium and phosphorus levels were also measured 24 hours later.

Demographic information such as age and gender were collected. Calcium and phosphorus levels were compared between the three Vit.D groups before surgery. This study was financially supported by Mashhad University of Medical Sciences (#4000784) and was approved by the local ethics committee (IR.MUMS. MEDICAL.REC.1400. 819).Data analysis was conducted using SPSS version 22 software. The normality of data distribution was determined by the Kolmogorov-Smirnov test. Mean±Standard deviation or median (interquartile range) were used for quantitative variables with normal and non-normal distribution, respectively. One-way ANOVA, or its non-parametric equivalent (Kruskal-Wallis test), was used to compare Vit.D groups. Chi-square or Fisher's exact test was used to compare the qualitative variables. A P<0.05 was considered statistically significant.

## Results

Among 100 patients who underwent TT from March to December 2022 and were enrolled in the present study, 81% were female. The mean age of the participants was 36.6±8.32 years 20 -54) (Table 1).

**Table 1:** Demographic and biochemical variables in all participants

	±Sd			
**Age**				
**Preoperative Vit. D**				
**Preoperative ca**				
**Preoperative P**				
**Preoperative Alb**				
**postoperative Ca**				
**postoperative P**				
**postoperative PTH**				

*Interquartile Range, Calcium (Ca), Phosphor (P), Albumin (Alb), Parathyroid Hormone (PTH)

Papillary thyroid carcinoma (58%) and multinodular goiter (37%) had the highest frequencies among our patients. Other causes include follicular neoplasm (2%), follicular thyroid carcinoma (2%), and Graves (1%). The mean Vit.D level before surgery was 26.9±16.89 ng/ml. As mentioned above, patients were assigned into three groups based on their Vit.D levels: 47% were in the normal Vit.D group, 32% in the insufficient group, and 21% in the deficient group.

There was no significant difference in age, gender, serum calcium, phosphorus, and albumin between these groups of Vit.D before surgery (Table 2,3).

 Also, no significant difference was found in postoperative calcium and phosphorus levels between these groups (Table 4).

**Table 2:** Comparison of demographic data in three groups of Vit.D

**Age (mean±SD)**					
**Sex N (%)**	Male				
	Female				

* One-Way Anova Test

** Chi-square test

**Table 3:** Comparison of preoperative laboratory data in three groups of Vit.D

**Preoperative Ca** **(mean ±SD)/** **median (IQR)⁎** *				
**Preoperative P** **(mean ±SD)/** **median (IQR)⁎** *				
**Preoperative Alb** **(mean ±SD)** **median (IQR)⁎** *				

*IQR=Interquartile Range

** One-Way Anova Test

Calcium (Ca), Phosphor (P), Albumin (Alb)

**Table 4:** Comparison of serum calcium and phosphorus 24 hours after surgery among Vit. D groups

**24 hours postoperative Ca ** **(mean ±SD)** **median (IQR)⁎** *	**8.80±1.35** 8.80(7.90-9.25)	**8.81±1.05** 8.81(8.229.27)	**9.00±1.00** 9.00(8.30-9.30)	0.356*
**24 hours postoperative P ** **(mean ±SD)** **median (IQR)** *	**3.75±0.75**	**3.66±0.64**	**3.77±0.58**	

*IQR=Interquartile Range

** Kruskal-Wallis’s Test

*** One-Way Anova Test

Calcium (ca), Phosphor (P)

The PTH level was measured before starting intravenous calcium in a few patients who needed calcium infusion based on their symptoms and signs, and/or serum calcium <8 mg/dl. Among 15 patients with laboratory or symptomatic hypocalcemia after surgery, seven were in the Vit.D deficiency group, three in the insufficient group, and five in the sufficient group. No significant difference was seen in the serum PTH levels between the three groups (P=0.596).

Subgroup analysis based on age distribution (below and above 40 years) showed no significant difference for either Vit.D level (P=0.151) or postoperative calcium (P=0.170) and other study variables. The same result was observed with patient subgroups based on the etiology of surgery.

## Discussion

Our study showed no significant difference in the preoperative serum calcium, phosphorus, and albumin between the three Vit.D groups. Similarly, no significant difference between calcium, phosphorus, and PTH levels was found 24 hours after TT between the three different Vit.D groups. 

Causative factors for hypocalcemia after TT have been investigated in several studies, which reflects the tendency to make more cost-effective and shorter hospitalization after TT (15, 16). Hypocalcemia after thyroidectomy can cause significant complications and prolong the length of hospitalization. Several factors, including autoimmune thyroid disease, substernal goiter, simultaneous thyroidectomy with parathyroidectomy, and the surgeon's experience, can increase the risk of hypocalcemia after total thyroidectomy (6). The loss of the compensatory role of PTH in secondary hyperparathyroidism due to Vit.D deficiency and the remaining need for bone restoration may increase the risk of hypocalcemia (17). However, the influence of Vit.D deficiency in predicting the risk of hypocalcemia after TTis unclear. Some studies have shown that Vit.D levels <20 ng/ml do not increase the risk of hypocalcemia after TT (18, 19). Hemmati et al. (20) and Soares CSP et al. (21) showed that serum levels of Vit.D before TT did not affect the occurrence of postoperative hypocalcemia. The results of our study with a larger sample size align with these investigations. In a prospective study by Godazandeh et al. (22) there was no significant relationship between hypocalcemia after TT and Vit.D deficiency before surgery. In this study, Vit.D levels <10 ng/ml were considered as Vit.D deficiency, but despite different criteria used to define Vit.D deficiency, the results by Godazandeh were similar to ours. Gurdeep (23) divided patients who underwent TT into two groups based on Vit.D levels (<20 ng/ml and ≥20 ng/ml) and showed that Vit.D level before TT did not affect the occurrence of hypocalcemia after surgery.

Evaluation of serum calcium, PTH, and Vit.D, 48 hours and six months after TT, was done by Karunakaran et al. (24) and showed that hypocalcemia was more common in patients with severe Vit.D deficiency. The presence of more patients with severe Vit.D deficiency (13%) in Karunakaran’s study versus our study (5%) may explain these different results. Vibhatavata P et al. (25) conducted a study to investigate the effect of Vit.D deficiency before surgery on the severity of hypocalcemia in patients with hypoparathyroidism following thyroidectomy. Patients were divided into two groups based on their Vit.D level before surgery. The Vit.D deficient group (<20 ng/ml) and the non-deficient group (≥20 ng/ml). Significant hypocalcemia was defined as calcium level <7.5 mg/dl. The serum calcium level was significantly lower in the Vit.D deficient group and the incidence of symptomatic hypocalcemia was significantly higher. The researchers pointed out that a preoperative Vit.D level <19.6 ng/ml can predict significant and symptomatic hypocalcemia in postoperative hypoparathyroidism with a sensitivity of 82-90% and a specificity of 70%. 

Unsal et al.'s study on 180 patients who were candidates for TT (26) showed that Vit.D deficiency is an independent factor in hypocalcemia after thyroidectomy. The difference in cut-off points for defining Vit.D deficiency and hypocalcemia can justify the difference in this study with our results. 

In two high sample-size investigations conducted by Choi EHE (27) and Carvalho (28), the incidence of postoperative hypocalcemia in the group with severe Vit.D deficiency was higher compared to the opposite group. Significant differences in the sample size of these studies may explain the opposite results of our study. 

A retrospective study by Qi Y et al. was conducted on 196 patients who underwent TT (29). This study divided patients into four groups based on their preoperative Vit.D levels. Patients with Vit.D levels >30 ng/ml were classified as the normal group. A Vit.D level between 20 and 30 ng/ml was defined as an insufficient level, a vitamin level between 20 and 10 ng/ml was defined as a Vit.D deficiency, and a level <10 ng/ml was defined as a severe Vit.D deficiency. Based on the results of this study, the incidence of postoperative hypocalcemia was higher in people with severe Vit.D deficiency. The results showed that Vit.D level <20 ng/ml and especially <10 ng/ml is an independent factor for postoperative hypocalcemia. In this study, postoperative PTH levels were lower in patients with Vit.D deficiency. However, in our study, the level of PTH in a few patients with Vit.D levels <10 ng/ml was in the normal range. This finding reflects the role of the high-volume surgeon in our study in preventing post-surgical hypoparathyroidism. 

Finally, the results of several studies (17,30,31), including two meta-analyses (32,33), are contrary to our results and have suggested Vit.D deficiency as one of the risk factors predicting the occurrence of post-thyroidectomy hypocalcemia. Ongoing contradictory results may explain the need for well-designed investigations into this dilemma.

## Conclusion

 Based on the results of the present study, Vit.D deficiency before TT is not related to the occurrence of hypocalcemia after surgery. Considering the conflicting results of previous studies, it is reasonable to conduct more research on this matter with similar designs and criteria for the definition of Vit.D deficiency.
